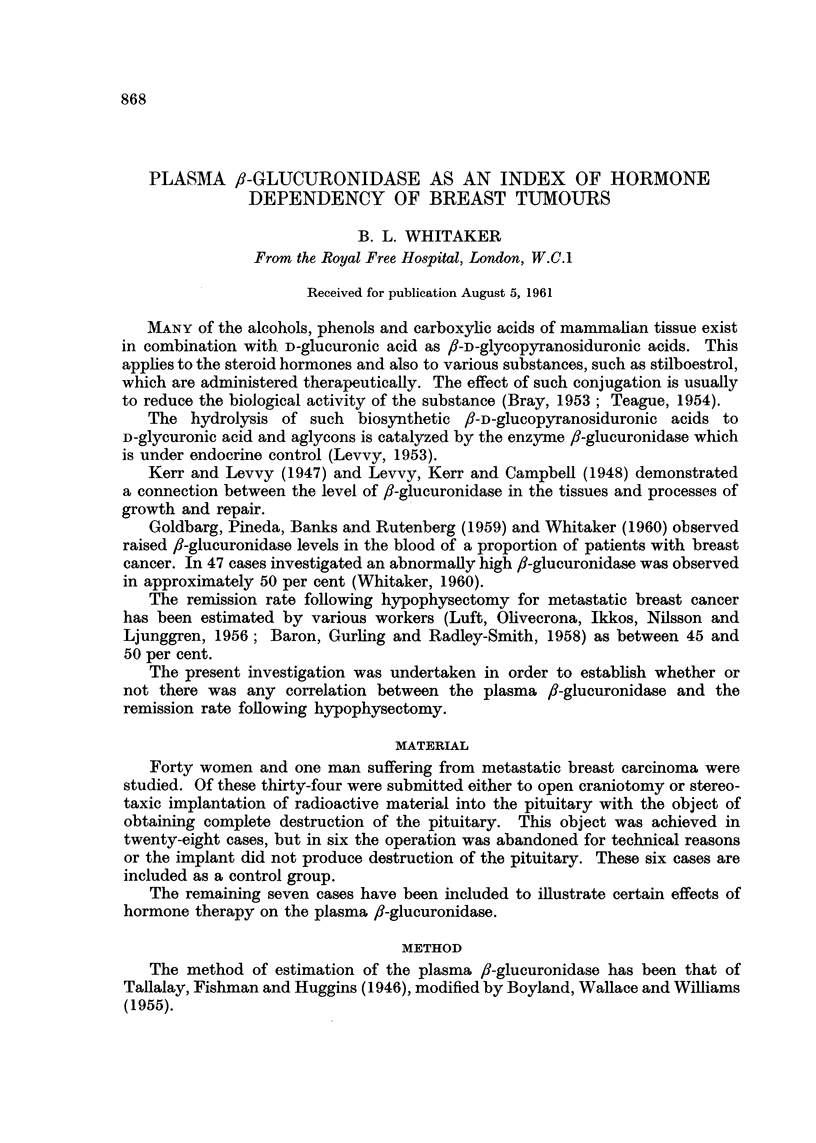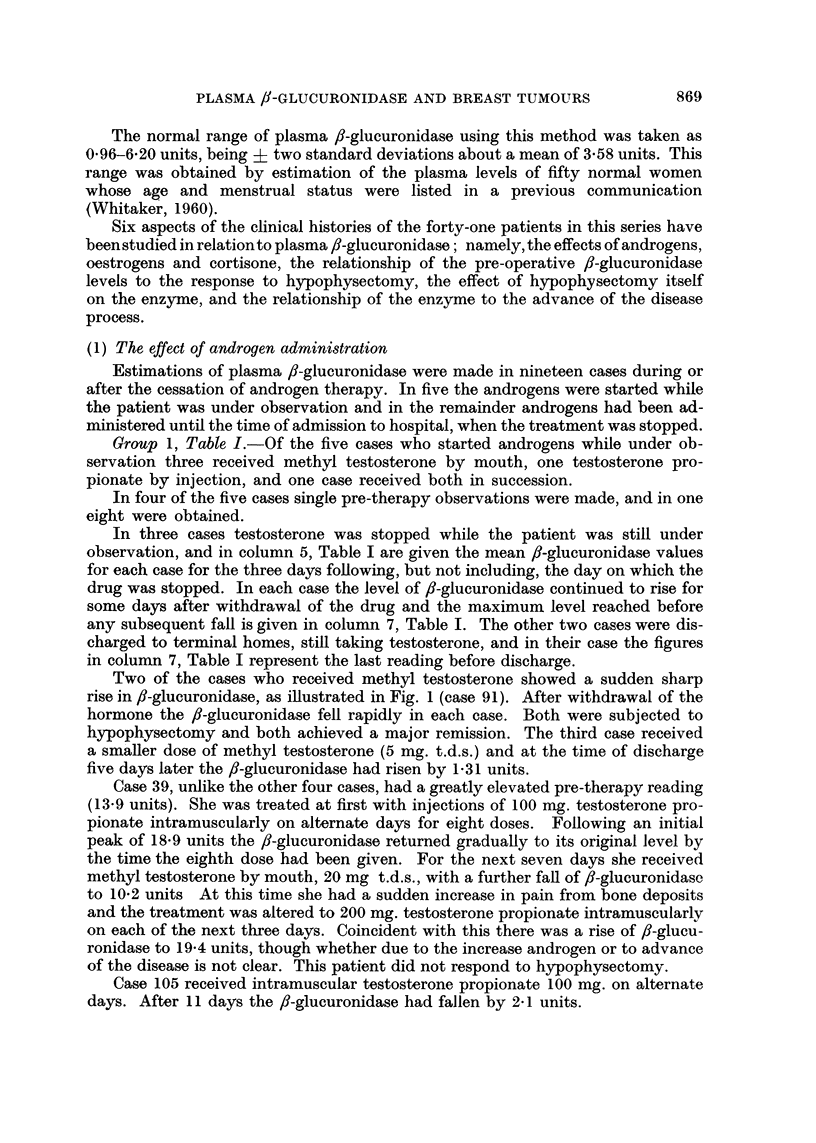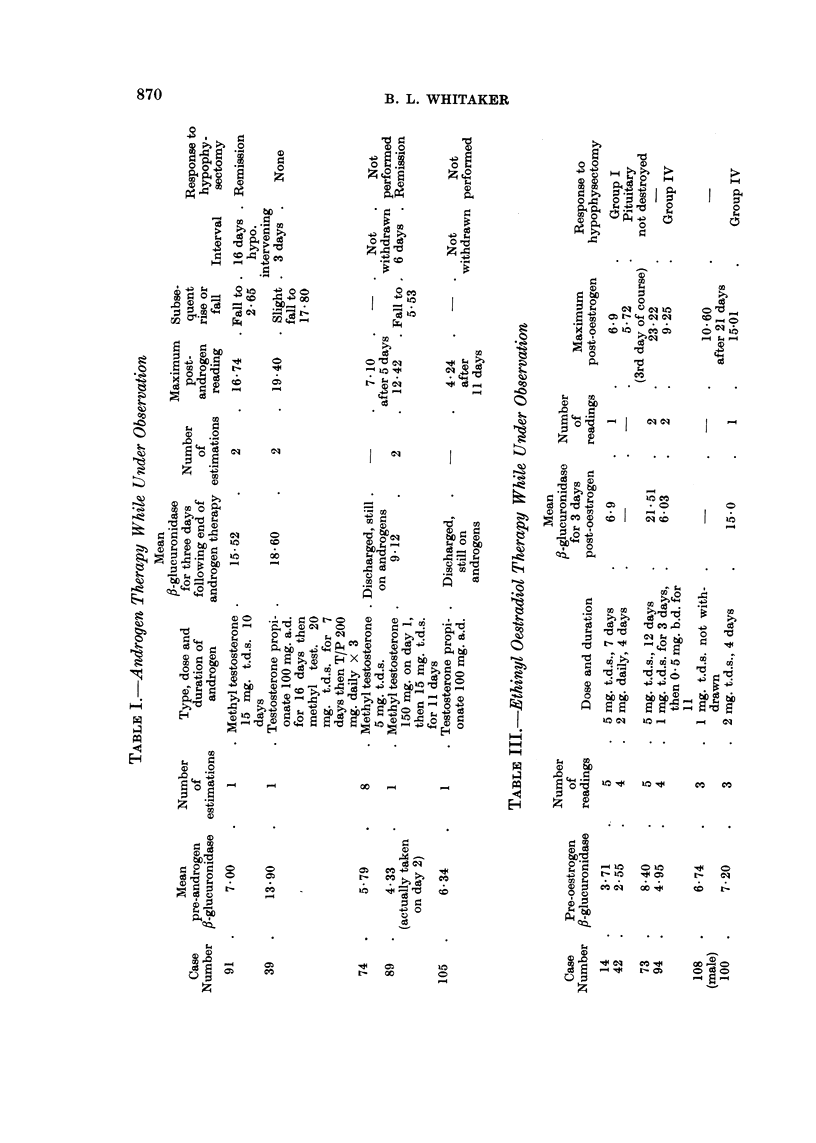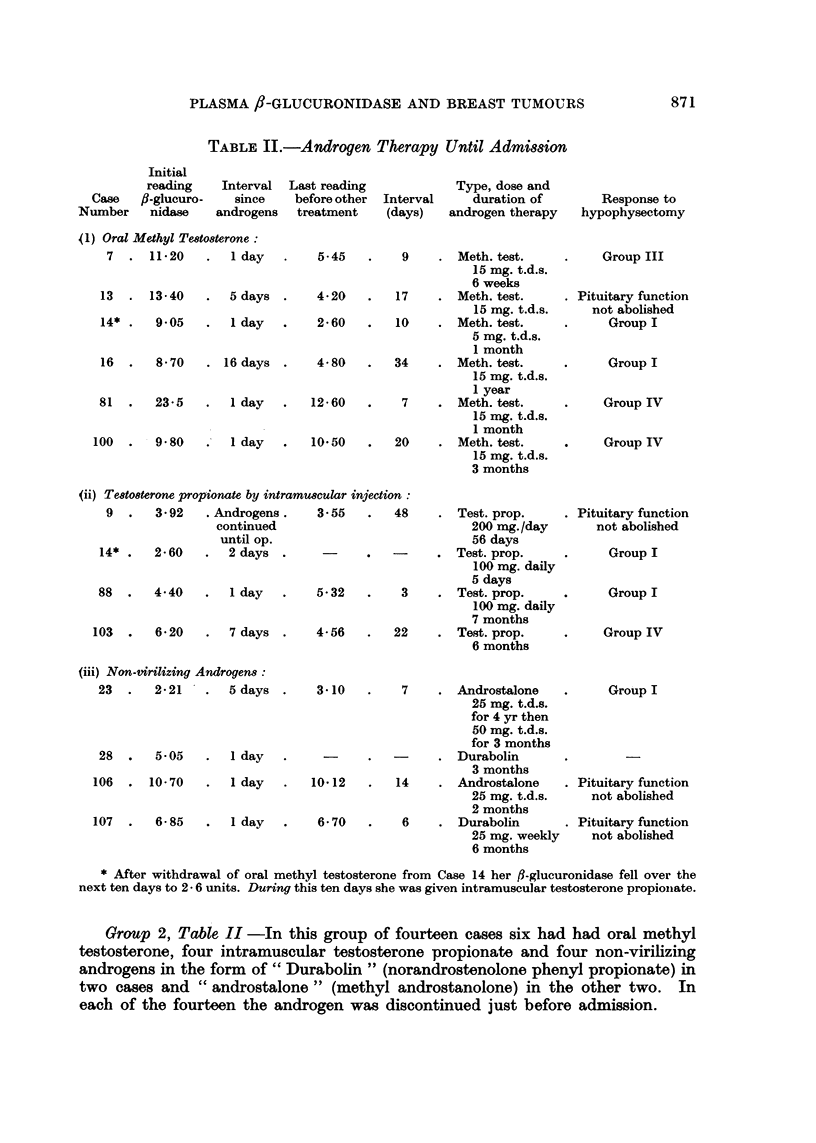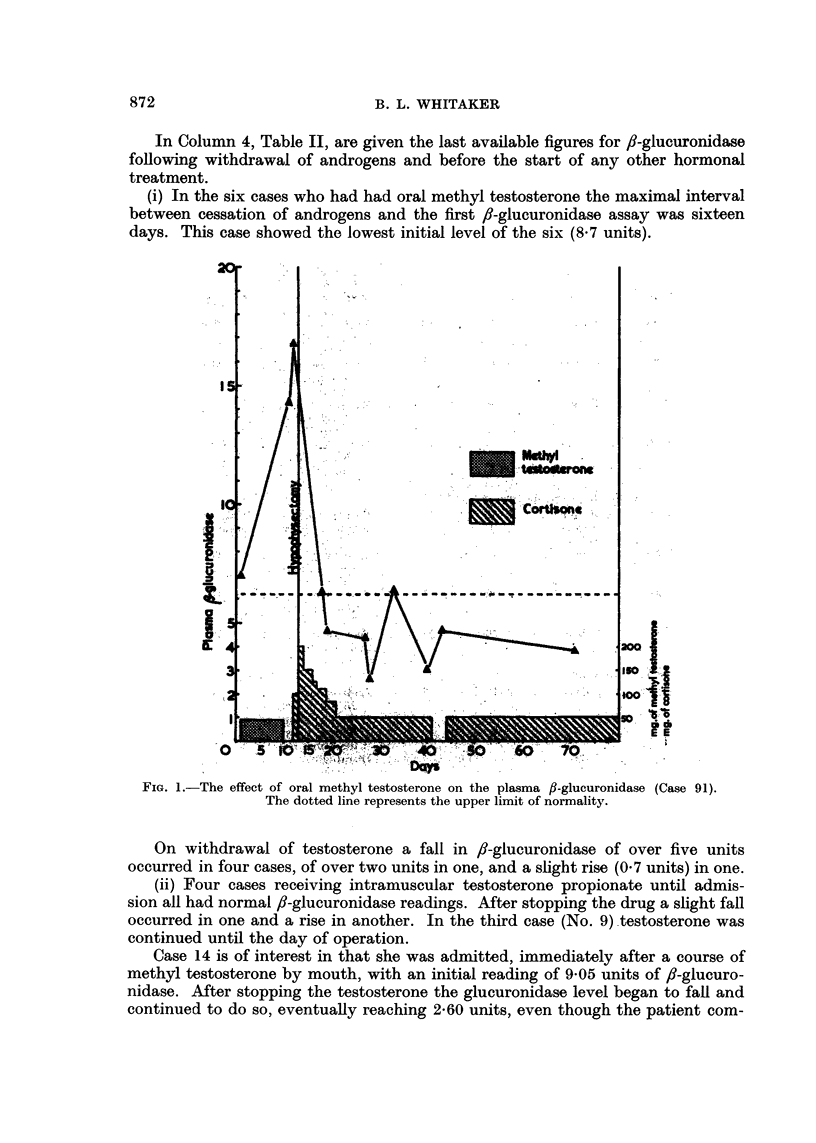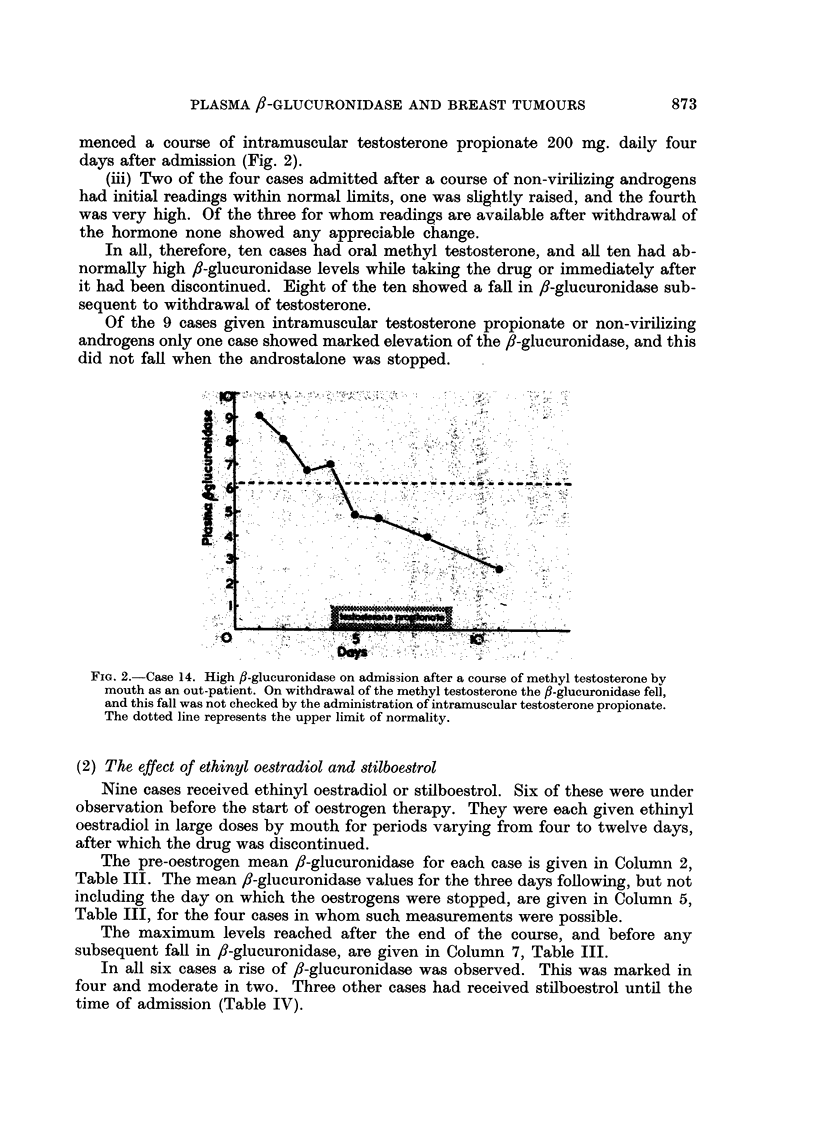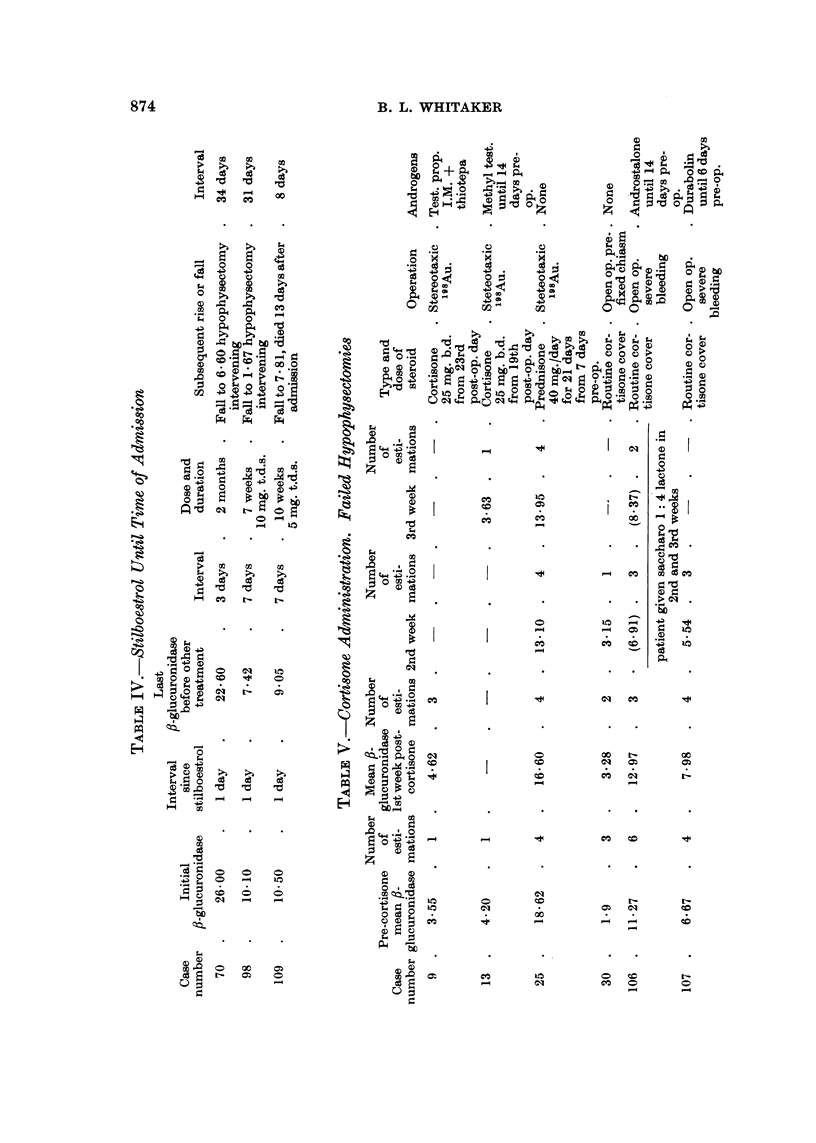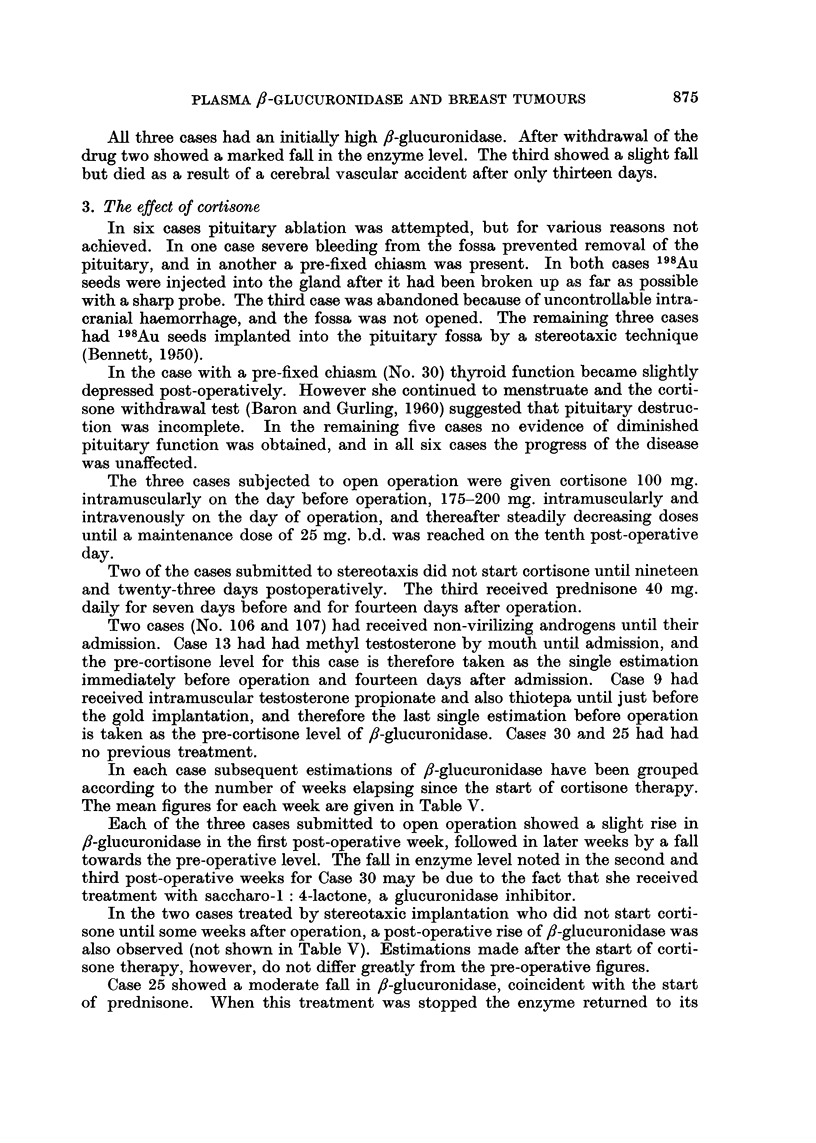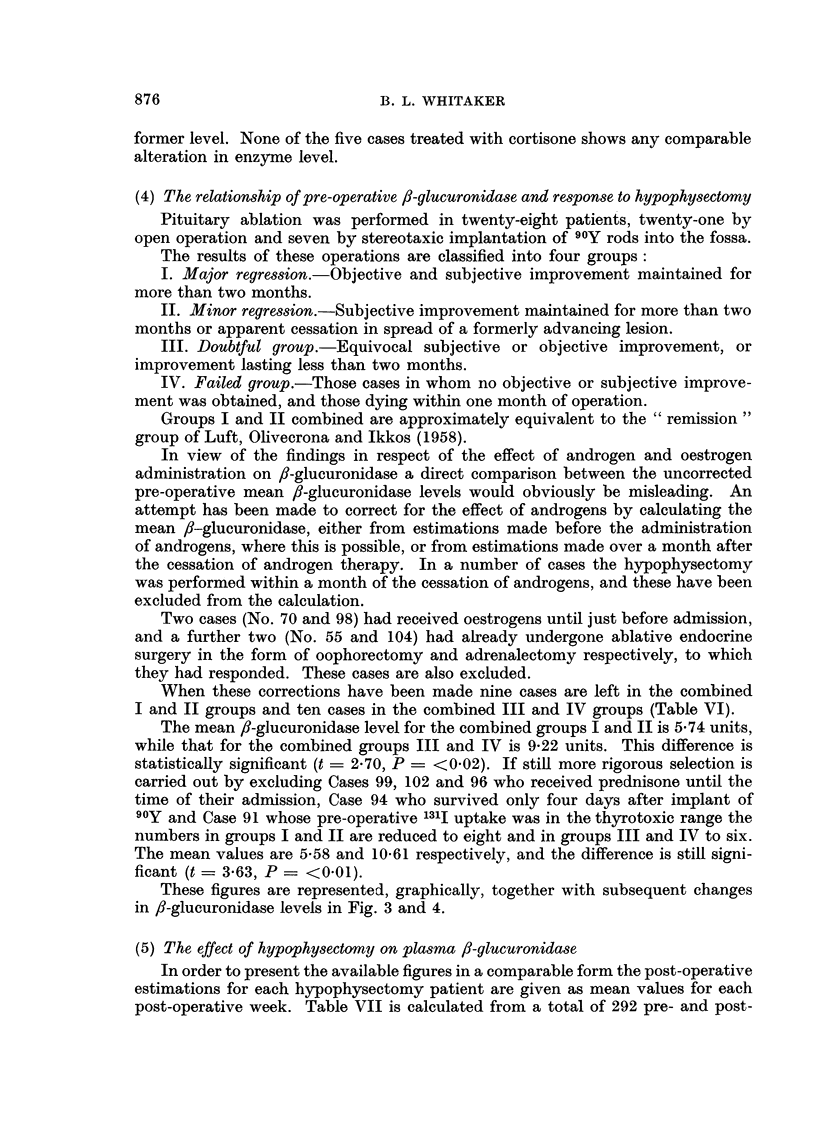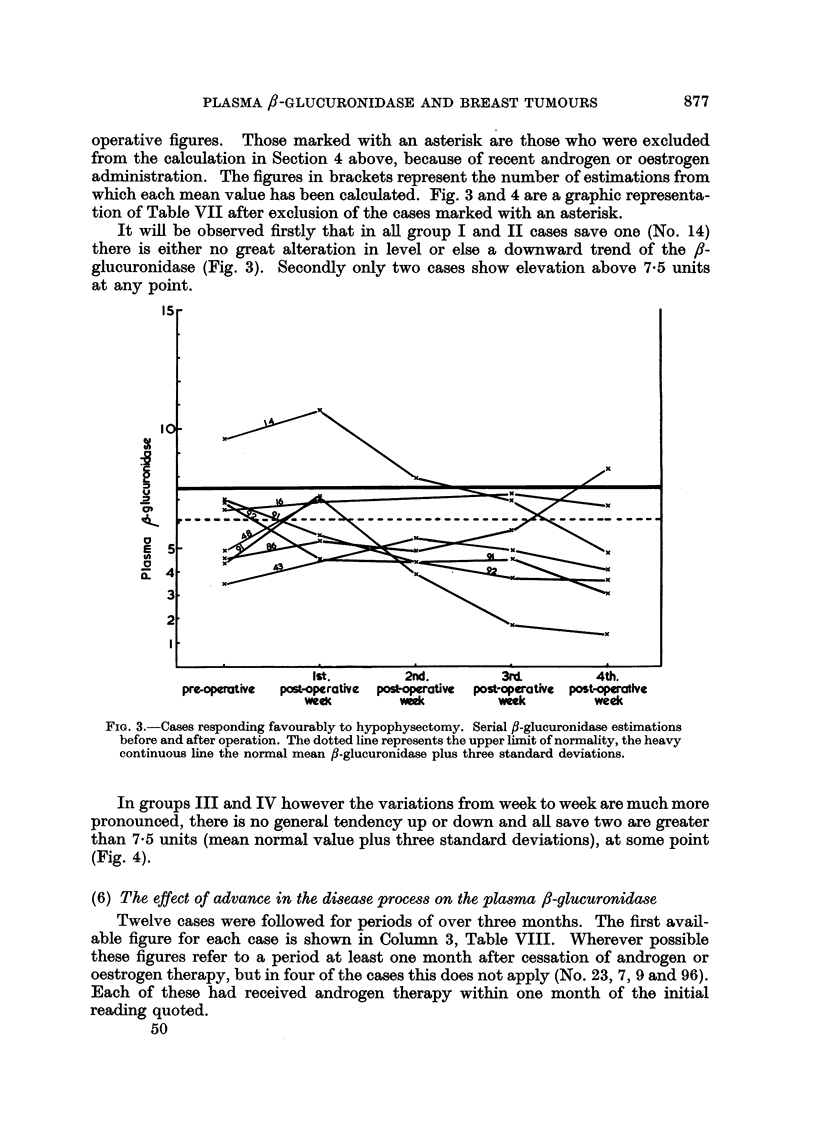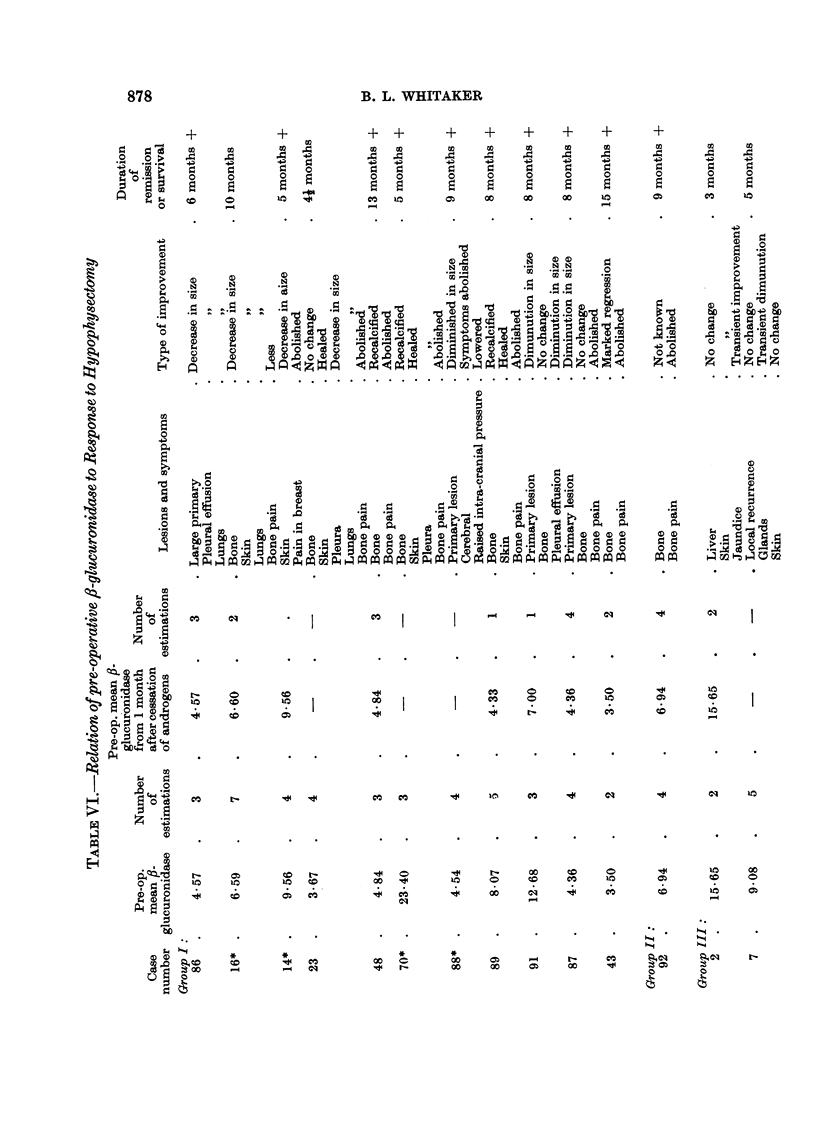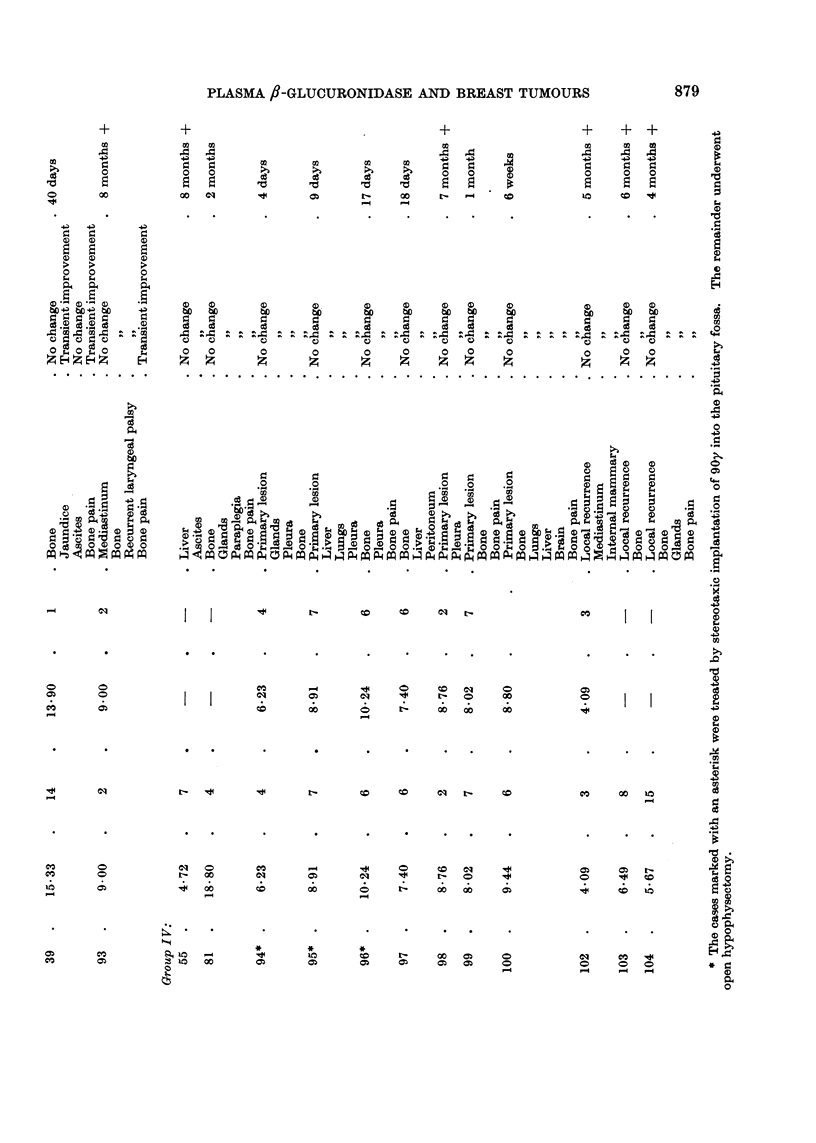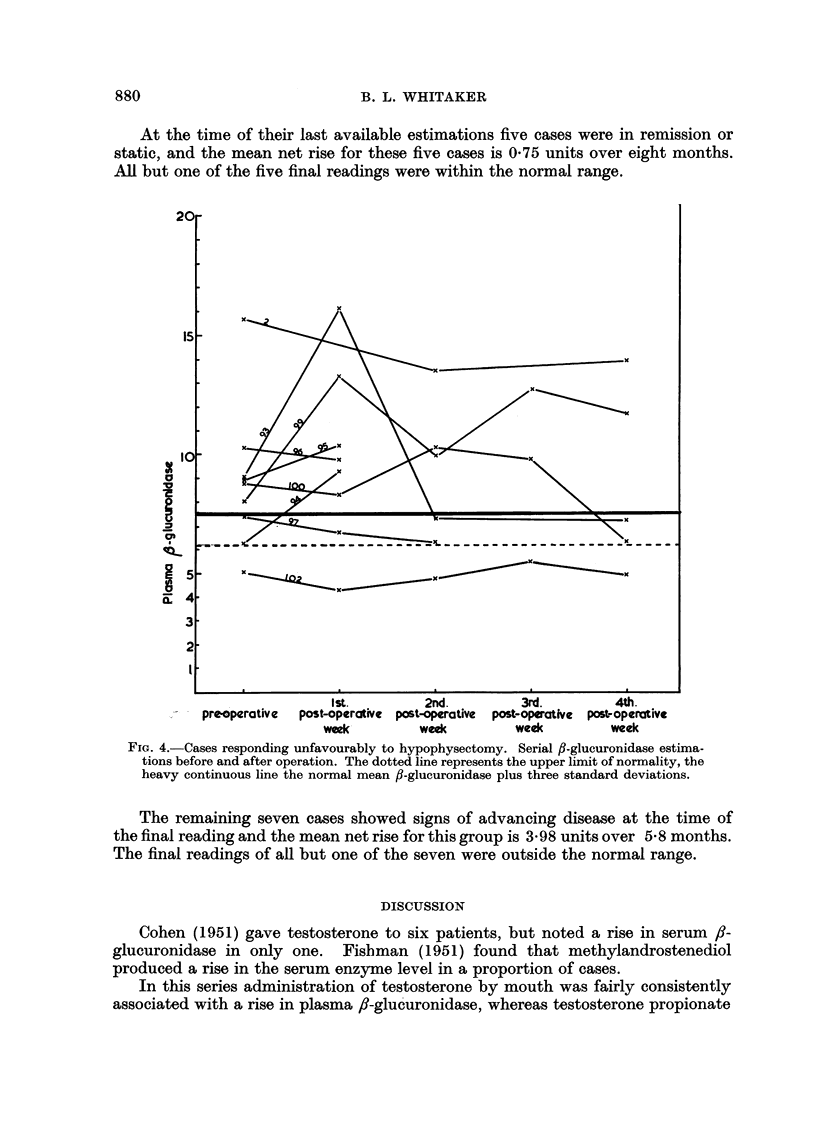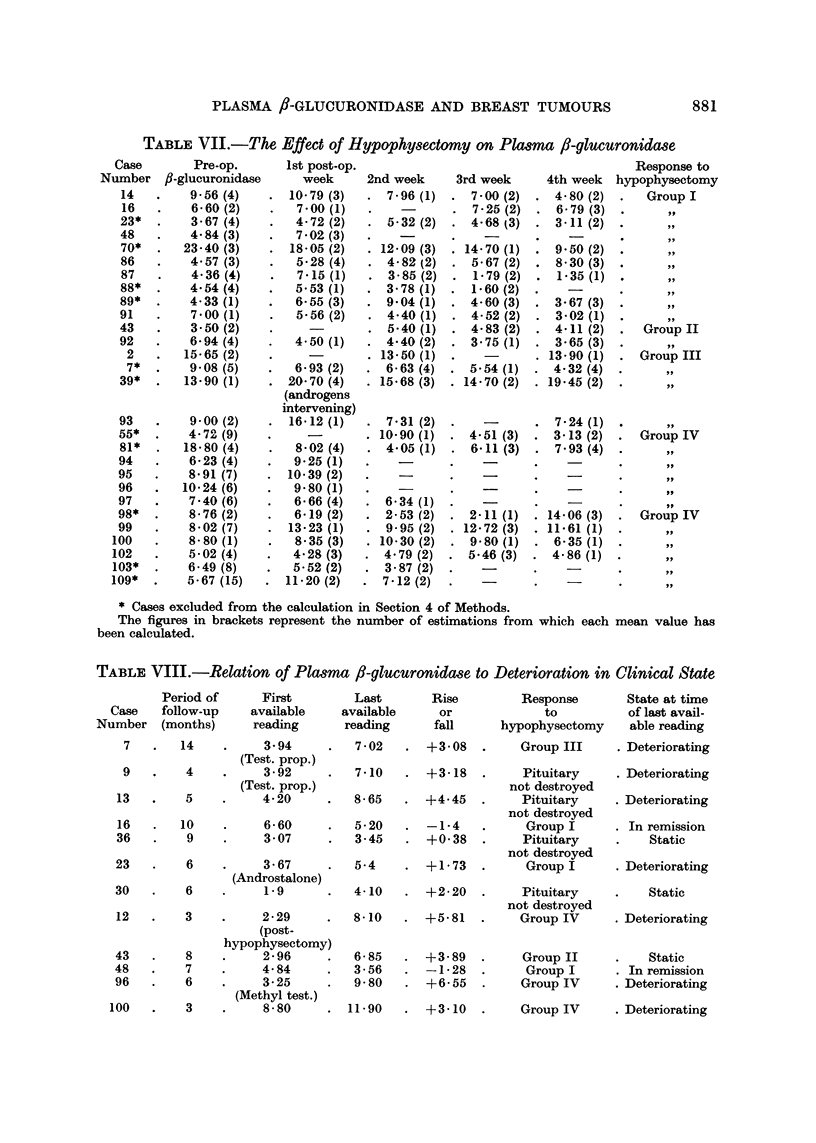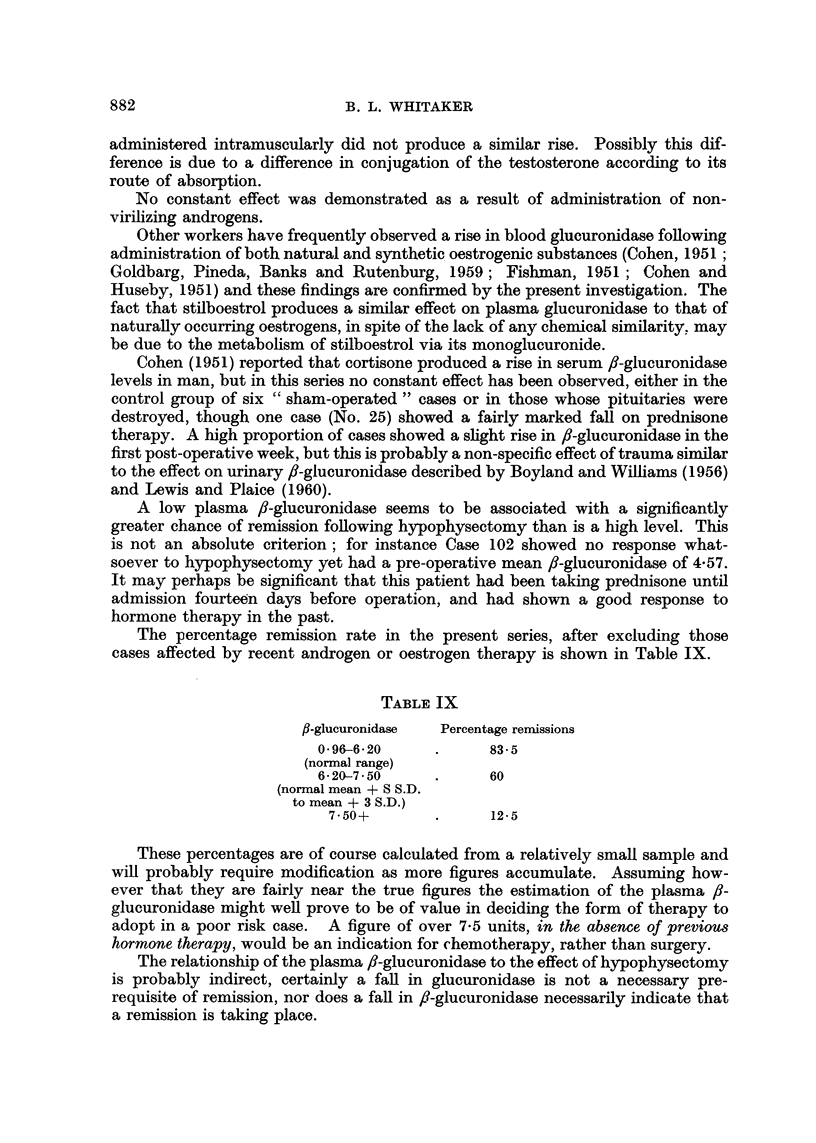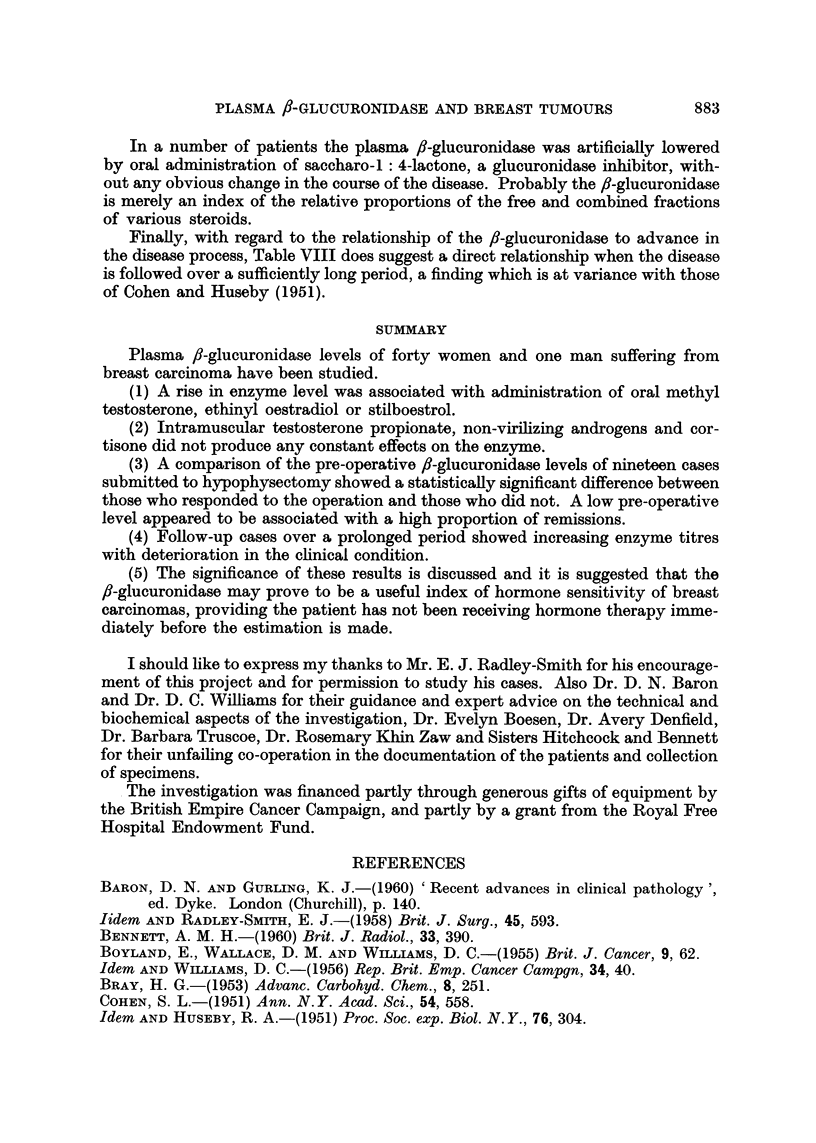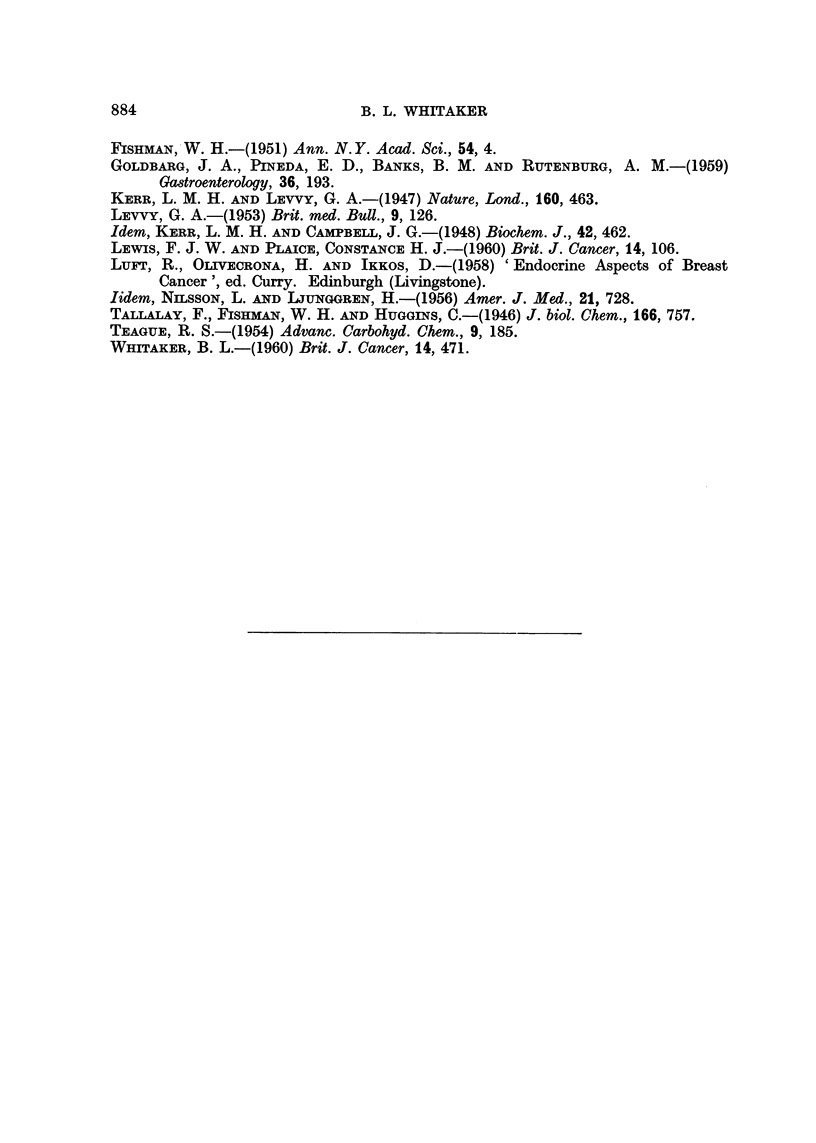# Plasma β-Glucuronidase as an Index of Hormone Dependency of Breast Tumours

**DOI:** 10.1038/bjc.1961.101

**Published:** 1961-12

**Authors:** B. L. Whitaker


### PLASMA β-GLUCURONIDASE AS AN INDEX OF HORMONE DEPENDENCY OF BREAST TUMOURS

B. L. WHITAKER

*From the Royal Free Hospital, London, W.C.1*

Received for publication August 5, 1961

MANY of the alcohols, phenols and carboxylic acids of mammalian tissue exist in combination with D-glucuronic acid as β-D-glycopyranosiduronic acids. This applies to the steroid hormones and also to various substances, such as stilboestrol, which are administered therapeutically. The effect of such conjugation is usually to reduce the biological activity of the substance (Bray, 1953 ; Teague, 1954).

The hydrolysis of such biosynthetic β-D-glucopyranosiduronic acids to D-glycuronic acid and aglycons is catalyzed by the enzyme β-glucuronidase which is under endocrine control (Levvy, 1953).

Kerr and Levvy (1947) and Levvy, Kerr and Campbell (1948) demonstrated a connection between the level of β-glucuronidase in the tissues and processes of growth and repair.

Goldbarg, Pineda, Banks and Rutenberg (1959) and Whitaker (1960) observed raised β-glucuronidase levels in the blood of a proportion of patients with breast cancer. In 47 cases investigated an abnormally high β-glucuronidase was observed in approximately 50 per cent (Whitaker, 1960).

The remission rate following hypophysectomy for metastatic breast cancer has been estimated by various workers (Luft, Olivecrona, Ikkos, Nilsson and Ljunggren, 1956 ; Baron, Gurling and Radley-Smith, 1958) as between 45 and 50 per cent.

The present investigation was undertaken in order to establish whether or not there was any correlation between the plasma β-glucuronidase and the remission rate following hypophysectomy.

### MATERIAL

Forty women and one man suffering from metastatic breast carcinoma were studied. Of these thirty-four were submitted either to open craniotomy or stereotaxic implantation of radioactive material into the pituitary with the object of obtaining complete destruction of the pituitary. This object was achieved in twenty-eight cases, but in six the operation was abandoned for technical reasons or the implant did not produce destruction of the pituitary. These six cases are included as a control group.

The remaining seven cases have been included to illustrate certain effects of hormone therapy on the plasma β-glucuronidase.

### METHOD

The method of estimation of the plasma β-glucuronidase has been that of Tallalay, Fishman and Huggins (1946), modified by Boyland, Wallace and Williams (1955).

The normal range of plasma $\beta$-glucuronidase using this method was taken as 0·96–6·20 units, being $\pm$ two standard deviations about a mean of 3·58 units. This range was obtained by estimation of the plasma levels of fifty normal women whose age and menstrual status were listed in a previous communication (Whitaker, 1960).

Six aspects of the clinical histories of the forty-one patients in this series have been studied in relation to plasma $\beta$-glucuronidase; namely, the effects of androgens, oestrogens and cortisone, the relationship of the pre-operative $\beta$-glucuronidase levels to the response to hypophysectomy, the effect of hypophysectomy itself on the enzyme, and the relationship of the enzyme to the advance of the disease process.

(1) *The effect of androgen administration*

Estimations of plasma $\beta$-glucuronidase were made in nineteen cases during or after the cessation of androgen therapy. In five the androgens were started while the patient was under observation and in the remainder androgens had been administered until the time of admission to hospital, when the treatment was stopped.

*Group* 1, *Table I.*—Of the five cases who started androgens while under observation three received methyl testosterone by mouth, one testosterone propionate by injection, and one case received both in succession.

In four of the five cases single pre-therapy observations were made, and in one eight were obtained.

In three cases testosterone was stopped while the patient was still under observation, and in column 5, Table I are given the mean $\beta$-glucuronidase values for each case for the three days following, but not including, the day on which the drug was stopped. In each case the level of $\beta$-glucuronidase continued to rise for some days after withdrawal of the drug and the maximum level reached before any subsequent fall is given in column 7, Table I. The other two cases were discharged to terminal homes, still taking testosterone, and in their case the figures in column 7, Table I represent the last reading before discharge.

Two of the cases who received methyl testosterone showed a sudden sharp rise in $\beta$-glucuronidase, as illustrated in Fig. 1 (case 91). After withdrawal of the hormone the $\beta$-glucuronidase fell rapidly in each case. Both were subjected to hypophysectomy and both achieved a major remission. The third case received a smaller dose of methyl testosterone (5 mg. t.d.s.) and at the time of discharge five days later the $\beta$-glucuronidase had risen by 1·31 units.

Case 39, unlike the other four cases, had a greatly elevated pre-therapy reading (13·9 units). She was treated at first with injections of 100 mg. testosterone propionate intramuscularly on alternate days for eight doses. Following an initial peak of 18·9 units the $\beta$-glucuronidase returned gradually to its original level by the time the eighth dose had been given. For the next seven days she received methyl testosterone by mouth, 20 mg t.d.s., with a further fall of $\beta$-glucuronidase to 10·2 units At this time she had a sudden increase in pain from bone deposits and the treatment was altered to 200 mg. testosterone propionate intramuscularly on each of the next three days. Coincident with this there was a rise of $\beta$-glucuronidase to 19·4 units, though whether due to the increase androgen or to advance of the disease is not clear. This patient did not respond to hypophysectomy.

Case 105 received intramuscular testosterone propionate 100 mg. on alternate days. After 11 days the $\beta$-glucuronidase had fallen by 2·1 units.

TABLE I.—*Androgen Therapy While Under Observation*

| Case Number | Mean pre-androgen β-glucuronidase | Number of estimations | Type, dose and duration of androgen | Mean β-glucuronidase for three days following end of androgen therapy | Number of estimations | Maximum post-androgen reading | Subsequent rise or fall | Interval | Response to hypophysectomy |
|---|---|---|---|---|---|---|---|---|---|
| 91 | 7·00 | 1 | Methyl testosterone 15 mg. t.d.s. 10 days | 15·52 | 2 | 16·74 | Fall to 2·65 | 16 days hypo. intervening | Remission |
| 39 | 13·90 | 1 | Testosterone propionate 100 mg. a.d. for 16 days then methyl test. 20 mg. t.d.s. for 7 days then T/P 200 mg. daily × 3 | 18·60 | 2 | 19·40 | Slight fall to 17·80 | 3 days | None |
| 74 | 5·79 | 8 | Methyl testosterone 5 mg. t.d.s. | Discharged, still on androgens | — | 7·10 after 5 days | — | Not withdrawn | Not performed |
| 89 | 4·33 (actually taken on day 2) | 1 | Methyl testosterone 150 mg. on day 1, then 15 mg. t.d.s. for 11 days | 9·12 | 2 | 12·42 | Fall to 5·53 | 6 days | Remission |
| 105 | 6·34 | 1 | Testosterone propionate 100 mg. a.d. | Discharged, still on androgens | — | 4·24 after 11 days | — | Not withdrawn | Not performed |

TABLE III.—*Ethinyl Oestradiol Therapy While Under Observation*

| Case Number | Pre-oestrogen β-glucuronidase | Number of readings | Dose and duration | Mean β-glucuronidase for 3 days post-oestrogen | Number of readings | Maximum post-oestrogen | Response to hypophysectomy |
|---|---|---|---|---|---|---|---|
| 14 | 3·71 | 5 | 5 mg. t.d.s., 7 days | 6·9 | 1 | 6·9 | Group I |
| 42 | 2·55 | 4 | 2 mg. daily, 4 days | — | — | 5·72 (3rd day of course) | Pituitary not destroyed |
| 73 | 8·40 | 5 | 5 mg. t.d.s., 12 days | 21·51 | 2 | 23·22 | — |
| 94 | 4·95 | 4 | 1 mg. t.d.s. for 3 days, then 0·5 mg. b.d. for 11 | 6·03 | 2 | 9·25 | Group IV |
| 108 (male) | 6·74 | 3 | 1 mg. t.d.s. not withdrawn | — | — | 10·60 after 21 days | — |
| 100 | 7·20 | 3 | 2 mg. t.d.s., 4 days | 15·0 | 1 | 15·01 | Group IV |

TABLE II.—*Androgen Therapy Until Admission*

| Case Number | Initial reading β-glucuronidase | Interval since androgens | Last reading before other treatment | Interval (days) | Type, dose and duration of androgen therapy | Response to hypophysectomy |
|---|---|---|---|---|---|---|
| (1) *Oral Methyl Testosterone :* | | | | | | |
| 7 | 11·20 | 1 day | 5·45 | 9 | Meth. test. 15 mg. t.d.s. 6 weeks | Group III |
| 13 | 13·40 | 5 days | 4·20 | 17 | Meth. test. 15 mg. t.d.s. | Pituitary function not abolished |
| 14* | 9·05 | 1 day | 2·60 | 10 | Meth. test. 5 mg. t.d.s. 1 month | Group I |
| 16 | 8·70 | 16 days | 4·80 | 34 | Meth. test. 15 mg. t.d.s. 1 year | Group I |
| 81 | 23·5 | 1 day | 12·60 | 7 | Meth. test. 15 mg. t.d.s. 1 month | Group IV |
| 100 | 9·80 | 1 day | 10·50 | 20 | Meth. test. 15 mg. t.d.s. 3 months | Group IV |
| (ii) *Testosterone propionate by intramuscular injection :* | | | | | | |
| 9 | 3·92 | Androgens continued until op. | 3·55 | 48 | Test. prop. 200 mg./day 56 days | Pituitary function not abolished |
| 14* | 2·60 | 2 days | — | — | Test. prop. 100 mg. daily 5 days | Group I |
| 88 | 4·40 | 1 day | 5·32 | 3 | Test. prop. 100 mg. daily 7 months | Group I |
| 103 | 6·20 | 7 days | 4·56 | 22 | Test. prop. 6 months | Group IV |
| (iii) *Non-virilizing Androgens :* | | | | | | |
| 23 | 2·21 | 5 days | 3·10 | 7 | Androstalone 25 mg. t.d.s. for 4 yr then 50 mg. t.d.s. for 3 months | Group I |
| 28 | 5·05 | 1 day | — | — | Durabolin 3 months | — |
| 106 | 10·70 | 1 day | 10·12 | 14 | Androstalone 25 mg. t.d.s. 2 months | Pituitary function not abolished |
| 107 | 6·85 | 1 day | 6·70 | 6 | Durabolin 25 mg. weekly 6 months | Pituitary function not abolished |

* After withdrawal of oral methyl testosterone from Case 14 her β-glucuronidase fell over the next ten days to 2·6 units. *During* this ten days she was given intramuscular testosterone propionate.

*Group* 2, *Table II* —In this group of fourteen cases six had had oral methyl testosterone, four intramuscular testosterone propionate and four non-virilizing androgens in the form of " Durabolin " (norandrostenolone phenyl propionate) in two cases and " androstalone " (methyl androstanolone) in the other two. In each of the fourteen the androgen was discontinued just before admission.

In Column 4, Table II, are given the last available figures for $\beta$-glucuronidase following withdrawal of androgens and before the start of any other hormonal treatment.

(i) In the six cases who had had oral methyl testosterone the maximal interval between cessation of androgens and the first $\beta$-glucuronidase assay was sixteen days. This case showed the lowest initial level of the six (8·7 units).

FIG. 1.—The effect of oral methyl testosterone on the plasma $\beta$-glucuronidase (Case 91). The dotted line represents the upper limit of normality.

On withdrawal of testosterone a fall in $\beta$-glucuronidase of over five units occurred in four cases, of over two units in one, and a slight rise (0·7 units) in one.

(ii) Four cases receiving intramuscular testosterone propionate until admission all had normal $\beta$-glucuronidase readings. After stopping the drug a slight fall occurred in one and a rise in another. In the third case (No. 9) testosterone was continued until the day of operation.

Case 14 is of interest in that she was admitted, immediately after a course of methyl testosterone by mouth, with an initial reading of 9·05 units of $\beta$-glucuronidase. After stopping the testosterone the glucuronidase level began to fall and continued to do so, eventually reaching 2·60 units, even though the patient commenced a course of intramuscular testosterone propionate 200 mg. daily four days after admission (Fig. 2).

(iii) Two of the four cases admitted after a course of non-virilizing androgens had initial readings within normal limits, one was slightly raised, and the fourth was very high. Of the three for whom readings are available after withdrawal of the hormone none showed any appreciable change.

In all, therefore, ten cases had oral methyl testosterone, and all ten had abnormally high β-glucuronidase levels while taking the drug or immediately after it had been discontinued. Eight of the ten showed a fall in β-glucuronidase subsequent to withdrawal of testosterone.

Of the 9 cases given intramuscular testosterone propionate or non-virilizing androgens only one case showed marked elevation of the β-glucuronidase, and this did not fall when the androstalone was stopped.

FIG. 2.—Case 14. High β-glucuronidase on admission after a course of methyl testosterone by mouth as an out-patient. On withdrawal of the methyl testosterone the β-glucuronidase fell, and this fall was not checked by the administration of intramuscular testosterone propionate. The dotted line represents the upper limit of normality.

(2) *The effect of ethinyl oestradiol and stilboestrol*

Nine cases received ethinyl oestradiol or stilboestrol. Six of these were under observation before the start of oestrogen therapy. They were each given ethinyl oestradiol in large doses by mouth for periods varying from four to twelve days, after which the drug was discontinued.

The pre-oestrogen mean β-glucuronidase for each case is given in Column 2, Table III. The mean β-glucuronidase values for the three days following, but not including the day on which the oestrogens were stopped, are given in Column 5, Table III, for the four cases in whom such measurements were possible.

The maximum levels reached after the end of the course, and before any subsequent fall in β-glucuronidase, are given in Column 7, Table III.

In all six cases a rise of β-glucuronidase was observed. This was marked in four and moderate in two. Three other cases had received stilboestrol until the time of admission (Table IV).

TABLE IV.—*Stilboestrol Until Time of Admission*

| Case number | Initial β-glucuronidase | Interval since stilboestrol | Last β-glucuronidase before other treatment | Interval | Dose and duration | Subsequent rise or fall | Interval |
|---|---|---|---|---|---|---|---|
| 70 | 26·00 | 1 day | 22·60 | 3 days | 2 months | Fall to 6·60 hypophysectomy intervening | 34 days |
| 98 | 10·10 | 1 day | 7·42 | 7 days | 7 weeks 10 mg. t.d.s. | Fall to 1·67 hypophysectomy intervening | 31 days |
| 109 | 10·50 | 1 day | 9·05 | 7 days | 10 weeks 5 mg. t.d.s. | Fall to 7·81, died 13 days after admission | 8 days |

TABLE V.—*Cortisone Administration. Failed Hypophysectomies*

| Case number | Pre-cortisone mean β-glucuronidase | Number of esti-mations | Mean β-glucuronidase 1st week post-cortisone | Number of esti-mations | 2nd week | Number of esti-mations | 3rd week | Number of esti-mations | Type and dose of steroid | Operation | Androgens |
|---|---|---|---|---|---|---|---|---|---|---|---|
| 9 | 3·55 | 1 | 4·62 | 3 | — | — | — | — | Cortisone 25 mg. b.d. from 23rd post-op. day | Stereotaxic $^{198}$Au. | Test. prop. I.M. + thiotepa |
| 13 | 4·20 | 1 | — | — | — | — | 3·63 | 1 | Cortisone 25 mg. b.d. from 19th post-op. day | Steteotaxic $^{198}$Au. | Methyl test. until 14 days pre-op. |
| 25 | 18·62 | 4 | 16·60 | 4 | 13·10 | 4 | 13·95 | 4 | Prednisone 40 mg./day for 21 days from 7 days pre-op. | Steteotaxic $^{198}$Au. | None |
| 30 | 1·9 | 3 | 3·28 | 2 | 3·15 | 1 | — | — | Routine cortisone cover | Open op. pre-fixed chiasm | None |
| 106 | 11·27 | 6 | 12·97 | 3 | (6·91) | 3 | (8·37) | 2 | Routine cortisone cover | Open op. severe bleeding | Androstalone until 14 days pre-op. |
| | | | | | patient given saccharo 1 : 4 lactone in 2nd and 3rd weeks | | | | | | |
| 107 | 6·67 | 4 | 7·98 | 4 | 5·54 | 3 | — | — | Routine cortisone cover | Open op. severe bleeding | Durabolin until 6 days pre-op. |

All three cases had an initially high β-glucuronidase. After withdrawal of the drug two showed a marked fall in the enzyme level. The third showed a slight fall but died as a result of a cerebral vascular accident after only thirteen days.

3. *The effect of cortisone*

In six cases pituitary ablation was attempted, but for various reasons not achieved. In one case severe bleeding from the fossa prevented removal of the pituitary, and in another a pre-fixed chiasm was present. In both cases $^{198}$Au seeds were injected into the gland after it had been broken up as far as possible with a sharp probe. The third case was abandoned because of uncontrollable intracranial haemorrhage, and the fossa was not opened. The remaining three cases had $^{198}$Au seeds implanted into the pituitary fossa by a stereotaxic technique (Bennett, 1950).

In the case with a pre-fixed chiasm (No. 30) thyroid function became slightly depressed post-operatively. However she continued to menstruate and the cortisone withdrawal test (Baron and Gurling, 1960) suggested that pituitary destruction was incomplete. In the remaining five cases no evidence of diminished pituitary function was obtained, and in all six cases the progress of the disease was unaffected.

The three cases subjected to open operation were given cortisone 100 mg. intramuscularly on the day before operation, 175–200 mg. intramuscularly and intravenously on the day of operation, and thereafter steadily decreasing doses until a maintenance dose of 25 mg. b.d. was reached on the tenth post-operative day.

Two of the cases submitted to stereotaxis did not start cortisone until nineteen and twenty-three days postoperatively. The third received prednisone 40 mg. daily for seven days before and for fourteen days after operation.

Two cases (No. 106 and 107) had received non-virilizing androgens until their admission. Case 13 had had methyl testosterone by mouth until admission, and the pre-cortisone level for this case is therefore taken as the single estimation immediately before operation and fourteen days after admission. Case 9 had received intramuscular testosterone propionate and also thiotepa until just before the gold implantation, and therefore the last single estimation before operation is taken as the pre-cortisone level of β-glucuronidase. Cases 30 and 25 had had no previous treatment.

In each case subsequent estimations of β-glucuronidase have been grouped according to the number of weeks elapsing since the start of cortisone therapy. The mean figures for each week are given in Table V.

Each of the three cases submitted to open operation showed a slight rise in β-glucuronidase in the first post-operative week, followed in later weeks by a fall towards the pre-operative level. The fall in enzyme level noted in the second and third post-operative weeks for Case 30 may be due to the fact that she received treatment with saccharo-1 : 4-lactone, a glucuronidase inhibitor.

In the two cases treated by stereotaxic implantation who did not start cortisone until some weeks after operation, a post-operative rise of β-glucuronidase was also observed (not shown in Table V). Estimations made after the start of cortisone therapy, however, do not differ greatly from the pre-operative figures.

Case 25 showed a moderate fall in β-glucuronidase, coincident with the start of prednisone. When this treatment was stopped the enzyme returned to its former level. None of the five cases treated with cortisone shows any comparable alteration in enzyme level.

(4) *The relationship of pre-operative β-glucuronidase and response to hypophysectomy*

Pituitary ablation was performed in twenty-eight patients, twenty-one by open operation and seven by stereotaxic implantation of $^{90}Y$ rods into the fossa.

The results of these operations are classified into four groups :

I. *Major regression.*—Objective and subjective improvement maintained for more than two months.

II. *Minor regression.*—Subjective improvement maintained for more than two months or apparent cessation in spread of a formerly advancing lesion.

III. *Doubtful group.*—Equivocal subjective or objective improvement, or improvement lasting less than two months.

IV. *Failed group.*—Those cases in whom no objective or subjective improvement was obtained, and those dying within one month of operation.

Groups I and II combined are approximately equivalent to the " remission " group of Luft, Olivecrona and Ikkos (1958).

In view of the findings in respect of the effect of androgen and oestrogen administration on β-glucuronidase a direct comparison between the uncorrected pre-operative mean β-glucuronidase levels would obviously be misleading. An attempt has been made to correct for the effect of androgens by calculating the mean β–glucuronidase, either from estimations made before the administration of androgens, where this is possible, or from estimations made over a month after the cessation of androgen therapy. In a number of cases the hypophysectomy was performed within a month of the cessation of androgens, and these have been excluded from the calculation.

Two cases (No. 70 and 98) had received oestrogens until just before admission, and a further two (No. 55 and 104) had already undergone ablative endocrine surgery in the form of oophorectomy and adrenalectomy respectively, to which they had responded. These cases are also excluded.

When these corrections have been made nine cases are left in the combined I and II groups and ten cases in the combined III and IV groups (Table VI).

The mean β-glucuronidase level for the combined groups I and II is 5·74 units, while that for the combined groups III and IV is 9·22 units. This difference is statistically significant ($t = 2{\cdot}70$, $P = <0{\cdot}02$). If still more rigorous selection is carried out by excluding Cases 99, 102 and 96 who received prednisone until the time of their admission, Case 94 who survived only four days after implant of $^{90}Y$ and Case 91 whose pre-operative $^{131}I$ uptake was in the thyrotoxic range the numbers in groups I and II are reduced to eight and in groups III and IV to six. The mean values are 5·58 and 10·61 respectively, and the difference is still significant ($t = 3{\cdot}63$, $P = <0{\cdot}01$).

These figures are represented, graphically, together with subsequent changes in β-glucuronidase levels in Fig. 3 and 4.

(5) *The effect of hypophysectomy on plasma β-glucuronidase*

In order to present the available figures in a comparable form the post-operative estimations for each hypophysectomy patient are given as mean values for each post-operative week. Table VII is calculated from a total of 292 pre- and postoperative figures. Those marked with an asterisk are those who were excluded from the calculation in Section 4 above, because of recent androgen or oestrogen administration. The figures in brackets represent the number of estimations from which each mean value has been calculated. Fig. 3 and 4 are a graphic representation of Table VII after exclusion of the cases marked with an asterisk.

It will be observed firstly that in all group I and II cases save one (No. 14) there is either no great alteration in level or else a downward trend of the β-glucuronidase (Fig. 3). Secondly only two cases show elevation above 7·5 units at any point.

FIG. 3.—Cases responding favourably to hypophysectomy. Serial β-glucuronidase estimations before and after operation. The dotted line represents the upper limit of normality, the heavy continuous line the normal mean β-glucuronidase plus three standard deviations.

In groups III and IV however the variations from week to week are much more pronounced, there is no general tendency up or down and all save two are greater than 7·5 units (mean normal value plus three standard deviations), at some point (Fig. 4).

(6) *The effect of advance in the disease process on the plasma β-glucuronidase*

Twelve cases were followed for periods of over three months. The first available figure for each case is shown in Column 3, Table VIII. Wherever possible these figures refer to a period at least one month after cessation of androgen or oestrogen therapy, but in four of the cases this does not apply (No. 23, 7, 9 and 96). Each of these had received androgen therapy within one month of the initial reading quoted.

TABLE VI.—*Relation of pre-operative β-glucuronidase to Response to Hypophysectomy*

| Case number | Pre-op. mean β-glucuronidase | Number of estimations | Pre-op. mean β-glucuronidase from 1 month after cessation of androgens | Number of estimations | Lesions and symptoms | Type of improvement | Duration of remission or survival |
|---|---|---|---|---|---|---|---|
| *Group I :* | | | | | | | |
| 86 | 4·57 | 3 | 4·57 | 3 | Large primary | Decrease in size | 6 months + |
| | | | | | Pleural effusion | ,, | |
| | | | | | Lungs | ,, | |
| 16* | 6·59 | 7 | 6·60 | 2 | Bone | Decrease in size | 10 months |
| | | | | | Skin | ,, | |
| | | | | | Lungs | ,, | |
| | | | | | Bone pain | Less | |
| 14* | 9·56 | 4 | 9·56 | . | Skin | Decrease in aize | 5 months + |
| | | | | | Pain in breast | Abolished | |
| 23 | 3·67 | 4 | — | — | Bone | No change | 4½ months |
| | | | | | Skin | Healed | |
| | | | | | Pleura | Decrease in size | |
| | | | | | Lungs | ,, | |
| | | | | | Bone pain | Abolished | |
| 48 | 4·84 | 3 | 4·84 | 3 | Bone | Recalcified | 13 months + |
| | | | | | Bone pain | Abolished | |
| 70* | 23·40 | 3 | — | — | Bone | Recalcified | 5 months + |
| | | | | | Skin | Healed | |
| | | | | | Pleura | ,, | |
| | | | | | Bone pain | Abolished | |
| 88* | 4·54 | 4 | — | — | Primary lesion | Diminished in size | 9 months + |
| | | | | | Cerebral | Symptoms abolished | |
| | | | | | Raised intra-cranial pressure | Lowered | |
| 89 | 8·07 | 5 | 4·33 | 1 | Bone | Recalcified | 8 months + |
| | | | | | Skin | Healed | |
| | | | | | Bone pain | Abolished | |
| 91 | 12·68 | 3 | 7·00 | 1 | Primary lesion | Dimunution in size | 8 months + |
| | | | | | Bone | No change | |
| | | | | | Pleural effusion | Diminution in size | |
| 87 | 4·36 | 4 | 4·36 | 4 | Primary lesion | Diminution in size | 8 months + |
| | | | | | Bone | No change | |
| | | | | | Bone pain | Abolished | |
| 43 | 3·50 | 2 | 3·50 | 2 | Bone | Marked regression | 15 months + |
| | | | | | Bone pain | Abolished | |
| *Group II :* | | | | | | | |
| 92 | 6·94 | 4 | 6·94 | 4 | Bone | Not known | 9 months + |
| | | | | | Bone pain | Abolished | |
| *Group III :* | | | | | | | |
| 2 | 15·65 | 2 | 15·65 | 2 | Liver | No change | 3 months |
| | | | | | Skin | ,, | |
| | | | | | Jaundice | Transient improvement | |
| 7 | 9·08 | 5 | — | — | Local recurrence | No change | 5 months |
| | | | | | Glands | Transient dimunution | |
| | | | | | Skin | No change | |

| | | | | | | | |
|---|---|---|---|---|---|---|---|
| 39 | 15·33 | 14 | 13·90 | 1 | Bone | No change | 40 days |
| | | | | | Jaundice | Transient improvement | |
| | | | | | Ascites | No change | |
| | | | | | Bone pain | Transient improvement | |
| 93 | 9·00 | 2 | 9·00 | 2 | Mediastinum | No change | 8 months + |
| | | | | | Bone | ,, | |
| | | | | | Recurrent laryngeal palsy | ,, | |
| | | | | | Bone pain | Transient improvement | |
| *Group IV:* | | | | | | | |
| 55 | 4·72 | 7 | — | — | Liver | No change | 8 months + |
| | | | | | Ascites | ,, | |
| 81 | 18·80 | 4 | — | — | Bone | No change | 2 months |
| | | | | | Glands | ,, | |
| | | | | | Paraplegia | ,, | |
| | | | | | Bone pain | ,, | |
| 94* | 6·23 | 4 | 6·23 | 4 | Primary lesion | No change | 4 days |
| | | | | | Glands | ,, | |
| | | | | | Pleura | ,, | |
| | | | | | Bone | ,, | |
| 95* | 8·91 | 7 | 8·91 | 7 | Primary lesion | No change | 9 days |
| | | | | | Liver | ,, | |
| | | | | | Lungs | ,, | |
| | | | | | Pleura | ,, | |
| 96* | 10·24 | 6 | 10·24 | 6 | Bone | No change | 17 days |
| | | | | | Pleura | ,, | |
| | | | | | Bone pain | ,, | |
| 97 | 7·40 | 6 | 7·40 | 6 | Bone | No change | 18 days |
| | | | | | Liver | ,, | |
| | | | | | Peritoneum | ,, | |
| 98 | 8·76 | 2 | 8·76 | 2 | Primary lesion | No change | 7 months + |
| | | | | | Pleura | ,, | |
| 99 | 8·02 | 7 | 8·02 | 7 | Primary lesion | No change | 1 month |
| | | | | | Bone | ,, | |
| | | | | | Bone pain | ,, | |
| 100 | 9·44 | 6 | 8·80 | | Primary lesion | No change | 6 weeks |
| | | | | | Bone | ,, | |
| | | | | | Lungs | ,, | |
| | | | | | Liver | ,, | |
| | | | | | Brain | ,, | |
| | | | | | Bone pain | ,, | |
| 102 | 4·09 | 3 | 4·09 | 3 | Local recurrence | No change | 5 months + |
| | | | | | Mediastinum | ,, | |
| | | | | | Internal mammary | ,, | |
| 103 | 6·49 | 8 | — | — | Local recurrence | No change | 6 months + |
| | | | | | Bone | ,, | |
| 104 | 5·67 | 15 | — | — | Local recurrence | No change | 4 months + |
| | | | | | Bone | ,, | |
| | | | | | Glands | ,, | |
| | | | | | Bone pain | ,, | |

* The cases marked with an asterisk were treated by stereotaxic implantation of 90γ into the pituitary fossa. The remainder underwent open hypophysectomy.

At the time of their last available estimations five cases were in remission or static, and the mean net rise for these five cases is 0·75 units over eight months. All but one of the five final readings were within the normal range.

FIG. 4.—Cases responding unfavourably to hypophysectomy. Serial β-glucuronidase estimations before and after operation. The dotted line represents the upper limit of normality, the heavy continuous line the normal mean β-glucuronidase plus three standard deviations.

The remaining seven cases showed signs of advancing disease at the time of the final reading and the mean net rise for this group is 3·98 units over 5·8 months. The final readings of all but one of the seven were outside the normal range.

### DISCUSSION

Cohen (1951) gave testosterone to six patients, but noted a rise in serum β-glucuronidase in only one. Fishman (1951) found that methylandrostenediol produced a rise in the serum enzyme level in a proportion of cases.

In this series administration of testosterone by mouth was fairly consistently associated with a rise in plasma β-glucuronidase, whereas testosterone propionate

TABLE VII.—*The Effect of Hypophysectomy on Plasma β-glucuronidase*

| Case Number | Pre-op. β-glucuronidase | 1st post-op. week | 2nd week | 3rd week | 4th week | Response to hypophysectomy |
|---|---|---|---|---|---|---|
| 14 | 9·56 (4) | 10·79 (3) | 7·96 (1) | 7·00 (2) | 4·80 (2) | Group I |
| 16 | 6·60 (2) | 7·00 (1) | — | 7·25 (2) | 6·79 (3) | ,, |
| 23* | 3·67 (4) | 4·72 (2) | 5·32 (2) | 4·68 (3) | 3·11 (2) | ,, |
| 48 | 4·84 (3) | 7·02 (3) | — | — | — | ,, |
| 70* | 23·40 (3) | 18·05 (2) | 12·09 (3) | 14·70 (1) | 9·50 (2) | ,, |
| 86 | 4·57 (3) | 5·28 (4) | 4·82 (2) | 5·67 (2) | 8·30 (3) | ,, |
| 87 | 4·36 (4) | 7·15 (1) | 3·85 (2) | 1·79 (2) | 1·35 (1) | ,, |
| 88* | 4·54 (4) | 5·53 (1) | 3·78 (1) | 1·60 (2) | — | ,, |
| 89* | 4·33 (1) | 6·55 (3) | 9·04 (1) | 4·60 (3) | 3·67 (3) | ,, |
| 91 | 7·00 (1) | 5·56 (2) | 4·40 (1) | 4·52 (2) | 3·02 (1) | ,, |
| 43 | 3·50 (2) | — | 5·40 (1) | 4·83 (2) | 4·11 (2) | Group II |
| 92 | 6·94 (4) | 4·50 (1) | 4·40 (2) | 3·75 (1) | 3·65 (3) | ,, |
| 2 | 15·65 (2) | — | 13·50 (1) | — | 13·90 (1) | Group III |
| 7* | 9·08 (5) | 6·93 (2) | 6·63 (4) | 5·54 (1) | 4·32 (4) | ,, |
| 39* | 13·90 (1) | 20·70 (4) (androgens intervening) | 15·68 (3) | 14·70 (2) | 19·45 (2) | ,, |
| 93 | 9·00 (2) | 16·12 (1) | 7·31 (2) | — | 7·24 (1) | ,, |
| 55* | 4·72 (9) | — | 10·90 (1) | 4·51 (3) | 3·13 (2) | Group IV |
| 81* | 18·80 (4) | 8·02 (4) | 4·05 (1) | 6·11 (3) | 7·93 (4) | ,, |
| 94 | 6·23 (4) | 9·25 (1) | — | — | — | ,, |
| 95 | 8·91 (7) | 10·39 (2) | — | — | — | ,, |
| 96 | 10·24 (6) | 9·80 (1) | — | — | — | ,, |
| 97 | 7·40 (6) | 6·66 (4) | 6·34 (1) | — | — | ,, |
| 98* | 8·76 (2) | 6·19 (2) | 2·53 (2) | 2·11 (1) | 14·06 (3) | Group IV |
| 99 | 8·02 (7) | 13·23 (1) | 9·95 (2) | 12·72 (3) | 11·61 (1) | ,, |
| 100 | 8·80 (1) | 8·35 (3) | 10·30 (2) | 9·80 (1) | 6·35 (1) | ,, |
| 102 | 5·02 (4) | 4·28 (3) | 4·79 (2) | 5·46 (3) | 4·86 (1) | ,, |
| 103* | 6·49 (8) | 5·52 (2) | 3·87 (2) | — | — | ,, |
| 109* | 5·67 (15) | 11·20 (2) | 7·12 (2) | — | — | ,, |

* Cases excluded from the calculation in Section 4 of Methods.

The figures in brackets represent the number of estimations from which each mean value has been calculated.

TABLE VIII.—*Relation of Plasma β-glucuronidase to Deterioration in Clinical State*

| Case Number | Period of follow-up (months) | First available reading | Last available reading | Rise or fall | Response to hypophysectomy | State at time of last available reading |
|---|---|---|---|---|---|---|
| 7 | 14 | 3·94 (Test. prop.) | 7·02 | +3·08 | Group III | Deteriorating |
| 9 | 4 | 3·92 (Test. prop.) | 7·10 | +3·18 | Pituitary not destroyed | Deteriorating |
| 13 | 5 | 4·20 | 8·65 | +4·45 | Pituitary not destroyed | Deteriorating |
| 16 | 10 | 6·60 | 5·20 | −1·4 | Group I | In remission |
| 36 | 9 | 3·07 | 3·45 | +0·38 | Pituitary not destroyed | Static |
| 23 | 6 | 3·67 (Androstalone) | 5·4 | +1·73 | Group I | Deteriorating |
| 30 | 6 | 1·9 | 4·10 | +2·20 | Pituitary not destroyed | Static |
| 12 | 3 | 2·29 (post-hypophysectomy) | 8·10 | +5·81 | Group IV | Deteriorating |
| 43 | 8 | 2·96 | 6·85 | +3·89 | Group II | Static |
| 48 | 7 | 4·84 | 3·56 | −1·28 | Group I | In remission |
| 96 | 6 | 3·25 (Methyl test.) | 9·80 | +6·55 | Group IV | Deteriorating |
| 100 | 3 | 8·80 | 11·90 | +3·10 | Group IV | Deteriorating |

administered intramuscularly did not produce a similar rise. Possibly this difference is due to a difference in conjugation of the testosterone according to its route of absorption.

No constant effect was demonstrated as a result of administration of non-virilizing androgens.

Other workers have frequently observed a rise in blood glucuronidase following administration of both natural and synthetic oestrogenic substances (Cohen, 1951; Goldbarg, Pineda, Banks and Rutenburg, 1959; Fishman, 1951; Cohen and Huseby, 1951) and these findings are confirmed by the present investigation. The fact that stilboestrol produces a similar effect on plasma glucuronidase to that of naturally occurring oestrogens, in spite of the lack of any chemical similarity, may be due to the metabolism of stilboestrol via its monoglucuronide.

Cohen (1951) reported that cortisone produced a rise in serum $\beta$-glucuronidase levels in man, but in this series no constant effect has been observed, either in the control group of six "sham-operated" cases or in those whose pituitaries were destroyed, though one case (No. 25) showed a fairly marked fall on prednisone therapy. A high proportion of cases showed a slight rise in $\beta$-glucuronidase in the first post-operative week, but this is probably a non-specific effect of trauma similar to the effect on urinary $\beta$-glucuronidase described by Boyland and Williams (1956) and Lewis and Plaice (1960).

A low plasma $\beta$-glucuronidase seems to be associated with a significantly greater chance of remission following hypophysectomy than is a high level. This is not an absolute criterion; for instance Case 102 showed no response whatsoever to hypophysectomy yet had a pre-operative mean $\beta$-glucuronidase of 4·57. It may perhaps be significant that this patient had been taking prednisone until admission fourteen days before operation, and had shown a good response to hormone therapy in the past.

The percentage remission rate in the present series, after excluding those cases affected by recent androgen or oestrogen therapy is shown in Table IX.

TABLE IX

| $\beta$-glucuronidase | Percentage remissions |
|---|---|
| 0·96–6·20 (normal range) | 83·5 |
| 6·20–7·50 (normal mean + S S.D. to mean + 3 S.D.) | 60 |
| 7·50+ | 12·5 |

These percentages are of course calculated from a relatively small sample and will probably require modification as more figures accumulate. Assuming however that they are fairly near the true figures the estimation of the plasma $\beta$-glucuronidase might well prove to be of value in deciding the form of therapy to adopt in a poor risk case. A figure of over 7·5 units, *in the absence of previous hormone therapy*, would be an indication for chemotherapy, rather than surgery.

The relationship of the plasma $\beta$-glucuronidase to the effect of hypophysectomy is probably indirect, certainly a fall in glucuronidase is not a necessary prerequisite of remission, nor does a fall in $\beta$-glucuronidase necessarily indicate that a remission is taking place.

In a number of patients the plasma β-glucuronidase was artificially lowered by oral administration of saccharo-1 : 4-lactone, a glucuronidase inhibitor, without any obvious change in the course of the disease. Probably the β-glucuronidase is merely an index of the relative proportions of the free and combined fractions of various steroids.

Finally, with regard to the relationship of the β-glucuronidase to advance in the disease process, Table VIII does suggest a direct relationship when the disease is followed over a sufficiently long period, a finding which is at variance with those of Cohen and Huseby (1951).

### SUMMARY

Plasma β-glucuronidase levels of forty women and one man suffering from breast carcinoma have been studied.

(1) A rise in enzyme level was associated with administration of oral methyl testosterone, ethinyl oestradiol or stilboestrol.

(2) Intramuscular testosterone propionate, non-virilizing androgens and cortisone did not produce any constant effects on the enzyme.

(3) A comparison of the pre-operative β-glucuronidase levels of nineteen cases submitted to hypophysectomy showed a statistically significant difference between those who responded to the operation and those who did not. A low pre-operative level appeared to be associated with a high proportion of remissions.

(4) Follow-up cases over a prolonged period showed increasing enzyme titres with deterioration in the clinical condition.

(5) The significance of these results is discussed and it is suggested that the β-glucuronidase may prove to be a useful index of hormone sensitivity of breast carcinomas, providing the patient has not been receiving hormone therapy immediately before the estimation is made.

I should like to express my thanks to Mr. E. J. Radley-Smith for his encouragement of this project and for permission to study his cases. Also Dr. D. N. Baron and Dr. D. C. Williams for their guidance and expert advice on the technical and biochemical aspects of the investigation, Dr. Evelyn Boesen, Dr. Avery Denfield, Dr. Barbara Truscoe, Dr. Rosemary Khin Zaw and Sisters Hitchcock and Bennett for their unfailing co-operation in the documentation of the patients and collection of specimens.

The investigation was financed partly through generous gifts of equipment by the British Empire Cancer Campaign, and partly by a grant from the Royal Free Hospital Endowment Fund.

### REFERENCES

Baron, D. N. and Gurling, K. J.—(1960) 'Recent advances in clinical pathology', ed. Dyke. London (Churchill), p. 140.

*Iidem* and Radley-Smith, E. J.—(1958) *Brit. J. Surg.*, **45**, 593.

Bennett, A. M. H.—(1960) *Brit. J. Radiol.*, **33**, 390.

Boyland, E., Wallace, D. M. and Williams, D. C.—(1955) *Brit. J. Cancer*, **9**, 62.

*Idem* and Williams, D. C.—(1956) *Rep. Brit. Emp. Cancer Campgn*, **34**, 40.

Bray, H. G.—(1953) *Advanc. Carbohyd. Chem.*, **8**, 251.

Cohen, S. L.—(1951) *Ann. N.Y. Acad. Sci.*, **54**, 558.

*Idem* and Huseby, R. A.—(1951) *Proc. Soc. exp. Biol. N.Y.*, **76**, 304.

FISHMAN, W. H.—(1951) *Ann. N.Y. Acad. Sci.*, **54**, 4.
GOLDBARG, J. A., PINEDA, E. D., BANKS, B. M. AND RUTENBURG, A. M.—(1959) *Gastroenterology*, **36**, 193.
KERR, L. M. H. AND LEVVY, G. A.—(1947) *Nature, Lond.*, **160**, 463.
LEVVY, G. A.—(1953) *Brit. med. Bull.*, **9**, 126.
*Idem*, KERR, L. M. H. AND CAMPBELL, J. G.—(1948) *Biochem. J.*, **42**, 462.
LEWIS, F. J. W. AND PLAICE, CONSTANCE H. J.—(1960) *Brit. J. Cancer*, **14**, 106.
LUFT, R., OLIVECRONA, H. AND IKKOS, D.—(1958) 'Endocrine Aspects of Breast Cancer', ed. Curry. Edinburgh (Livingstone).
*Iidem*, NILSSON, L. AND LJUNGGREN, H.—(1956) *Amer. J. Med.*, **21**, 728.
TALLALAY, F., FISHMAN, W. H. AND HUGGINS, C.—(1946) *J. biol. Chem.*, **166**, 757.
TEAGUE, R. S.—(1954) *Advanc. Carbohyd. Chem.*, **9**, 185.
WHITAKER, B. L.—(1960) *Brit. J. Cancer*, **14**, 471.